# Supplementary material for: Structure of anellovirus-like particles reveal a mechanism for immune evasion
Source: Nat Commun. 2024 Aug 22;15:7219. doi: 10.1038/s41467-024-51064-8 (PMC11341859; doi:10.1038/s41467-024-51064-8)
Supplement: Supplementary file 1 — Supplementary Information [file 41467_2024_51064_MOESM1_ESM.pdf]

Supporting Information

Supplementary Table 1. Cryo-EM data collection, refinement and validation statistics of LY1 ΔARM ORF1 fragment virus-like particle.

	LY1 ΔARM fragment virus-like particle
**Data collection and processing**	
Magnification	150000x
Voltage (kV)	200
Electron exposure (e–/Å^2)	1.31
Defocus range (μm)	-0.5~ -2.5
Pixel size (Å)	0.923
Symmetry imposed	I1
Initial particle images (no.)	58391
Final particle images (no.)	6271
Map resolution (Å)	3.98/0.143
FSC threshold	
Map resolution range (Å)	3.9-6
**Refinement**	
Model resolution (Å)	3.98/0.143
FSC threshold	
Model resolution range (Å)	3.9-6
Map sharpening *B* factor (Å^2)	-152.851
Model composition	253440/30900/0
Non-hydrogen atoms	
Protein residues	
Ligands	
B factors (Å^2)	30.00/243.72/133.17
Protein	
Ligand	
R.m.s. deviations	0.007/1.282
Bond lengths (Å)	
Bond angles (°)	
Validation	2.36/25.43/0
MolProbity score	
Clashscore	
Poor rotamers (%)	
Ramachandran plot	92.6/6.62/0.78
Favored (%)	
Allowed (%)	
Disallowed (%)	
CC (mask)	0.81

Supplementary Table 2. Cryo-EM data collection, refinement and validation statistics of LY1 ΔC-Term virus-like particle.

	LY1 ΔC-Term virus-like particle
**Data collection and processing**	
Magnification	105000x
Voltage (kV)	300
Electron exposure (e–/Å^2)	1.4
Defocus range (μm)	-1.2~ -1.6
Pixel size (Å)	0.834
Symmetry imposed	I1
Final particle images (no.)	22743
Map resolution (Å)<br>FSC threshold	2.69/0.143
Map resolution range (Å)	2.69-4.2
**Refinement**	
Model resolution (Å)<br>FSC threshold	2.83/0.143
Model resolution range (Å)	2.8-4.2
Map sharpening *B* factor (Å^2)	83.3
Model composition<br>Non-hydrogen atoms<br>Protein residues<br>Ligands	253440/30900/0
B factors (Å^2)<br>Protein<br>Ligand	30.00/243.72/133.17
R.m.s. deviations<br>Bond lengths (Å)<br>Bond angles (°)	0.012/1.291
Validation<br>MolProbity score<br>Clashscore<br>Poor rotamers (%)	1.15/22.43/0
Ramachandran plot<br>Favored (%)<br>Allowed (%)<br>Disallowed (%)	99.03/0.97/0
CC (mask)	0.63

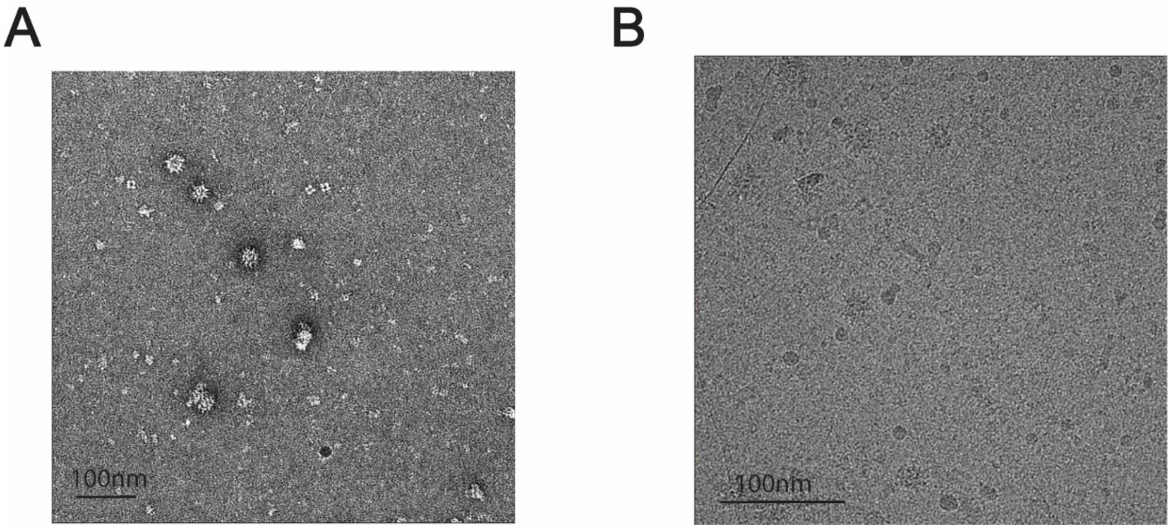

Supplementary Fig. 1. Representative micrographs of LY1 ΔARM fragment virus-like particle. A. and **B.** are representative negative-stained and cryo-EM micrographs for LY1 ΔARM, respectively.

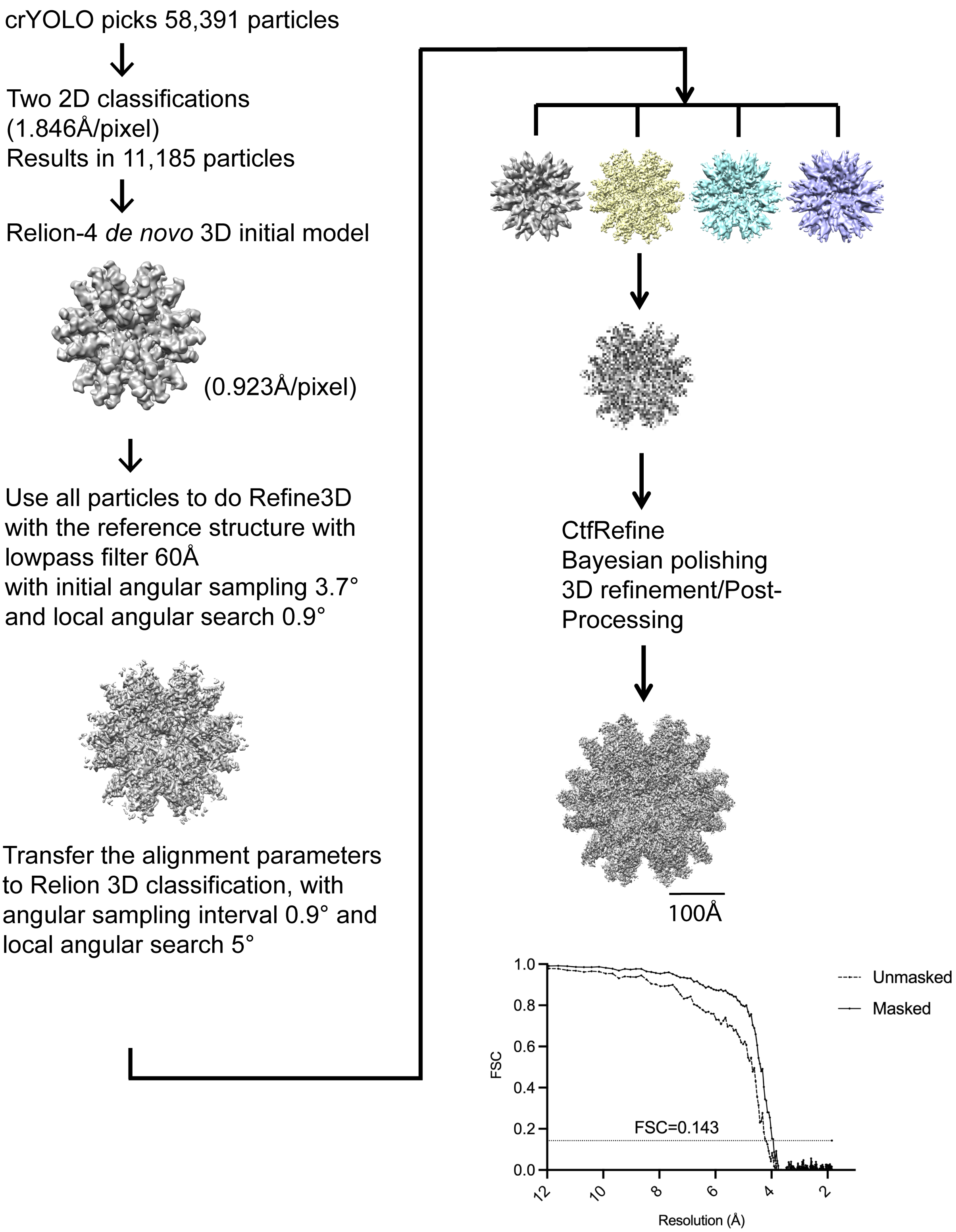

Supplementary Fig. 2. Image processing work-flow of the LY1 ΔARM fragment virus-like particle.

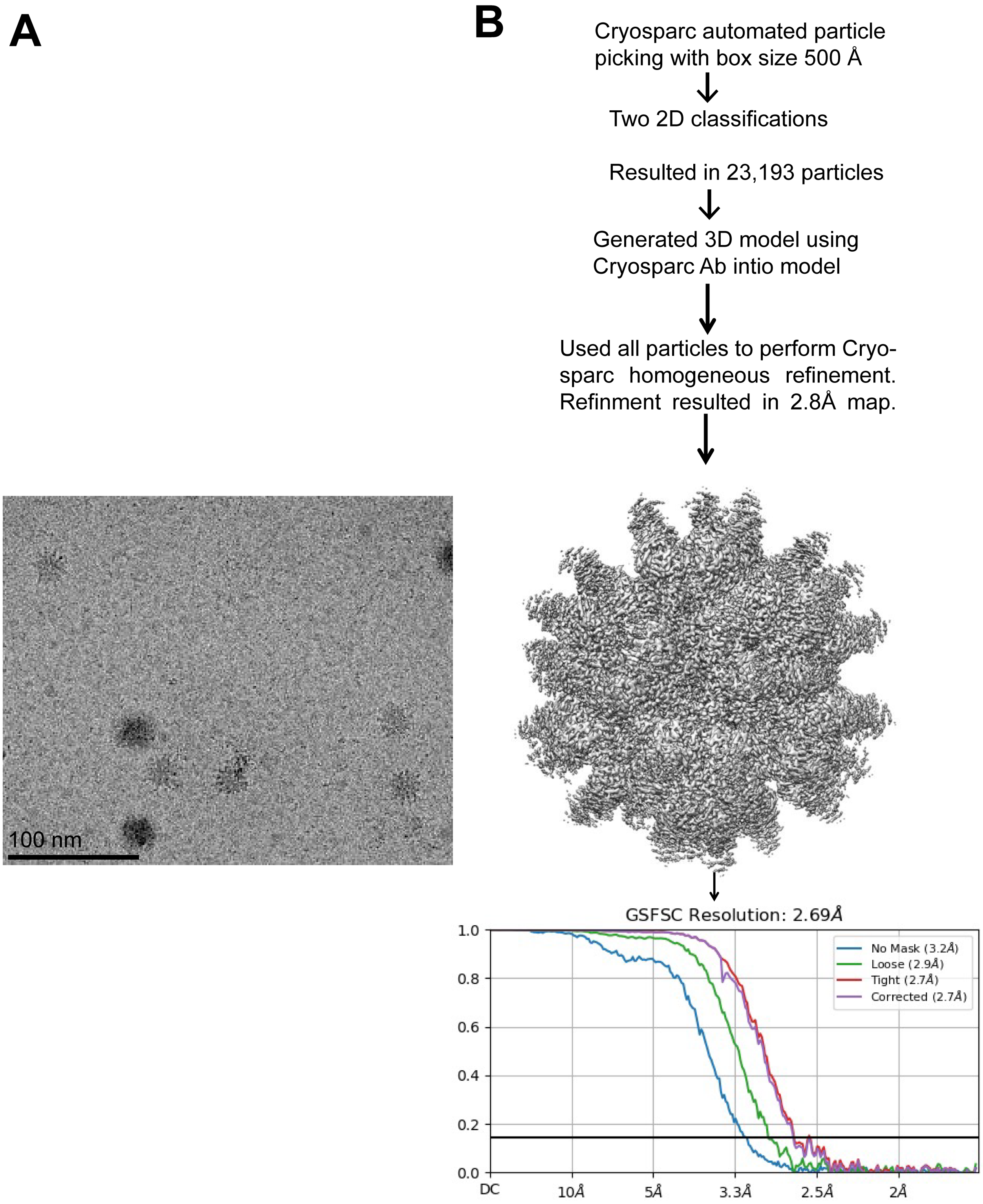

Supplementary Fig. 3. Image processing work-flow of the LY1 ΔC-term virus-like particle: **A.** cryo-EM micrographs for LY1 ΔC-term virus-like particle. **B.** Image processing work-flow of LY1 ΔC-term virus-like particle

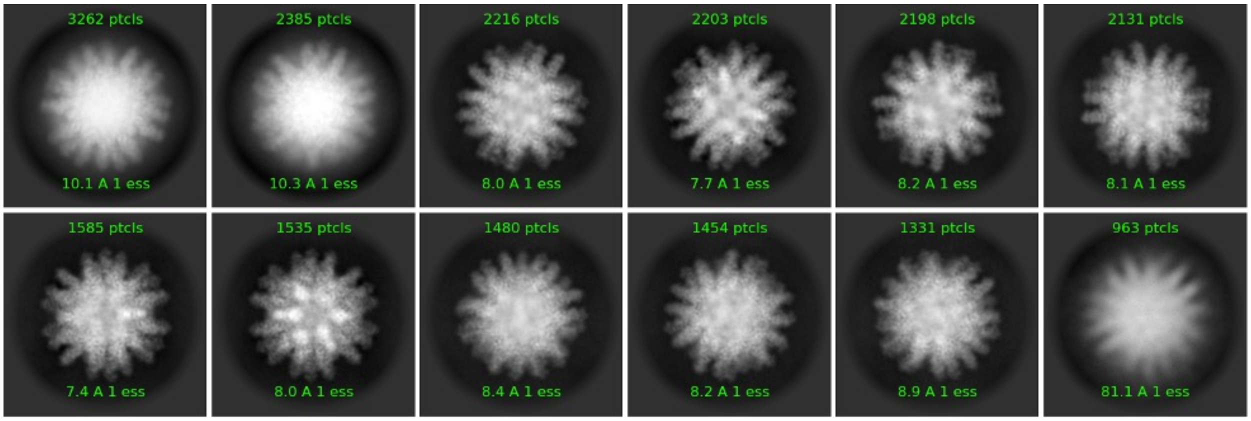

Supplementary Fig. 4. Representative 2D class averages of LY1 ΔC-term virus-like particle. Subset of the final round of 2D class averaging for LY1-ΔC-term virus-like particle sample obtained from cryoSPARC3.3. Box size is 500 Å.

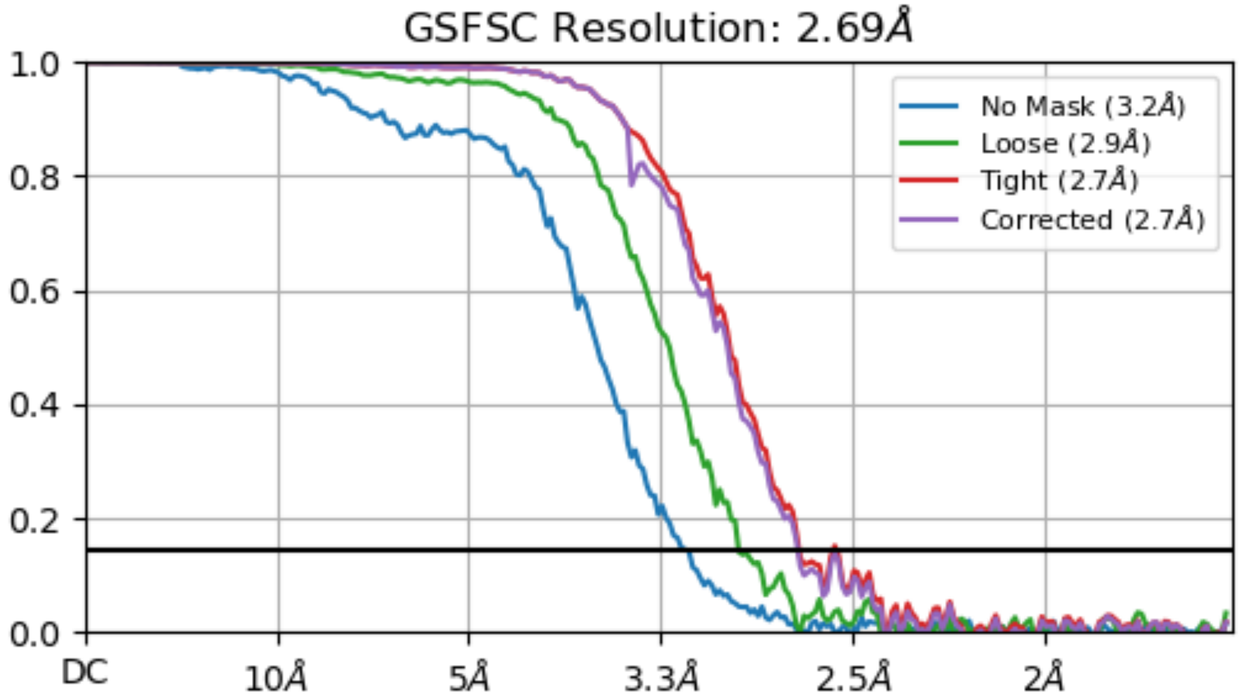

Supplementary Fig. 5. "Gold standard" FSC curves of the final 3D refinement for LY1 ΔC-Term virus-like particle. CryoSPARC 3.3 analysis results for different types of masks. The resolution of the final map was calculated based on a Fourier shell correlation (FSC) of 0.143.

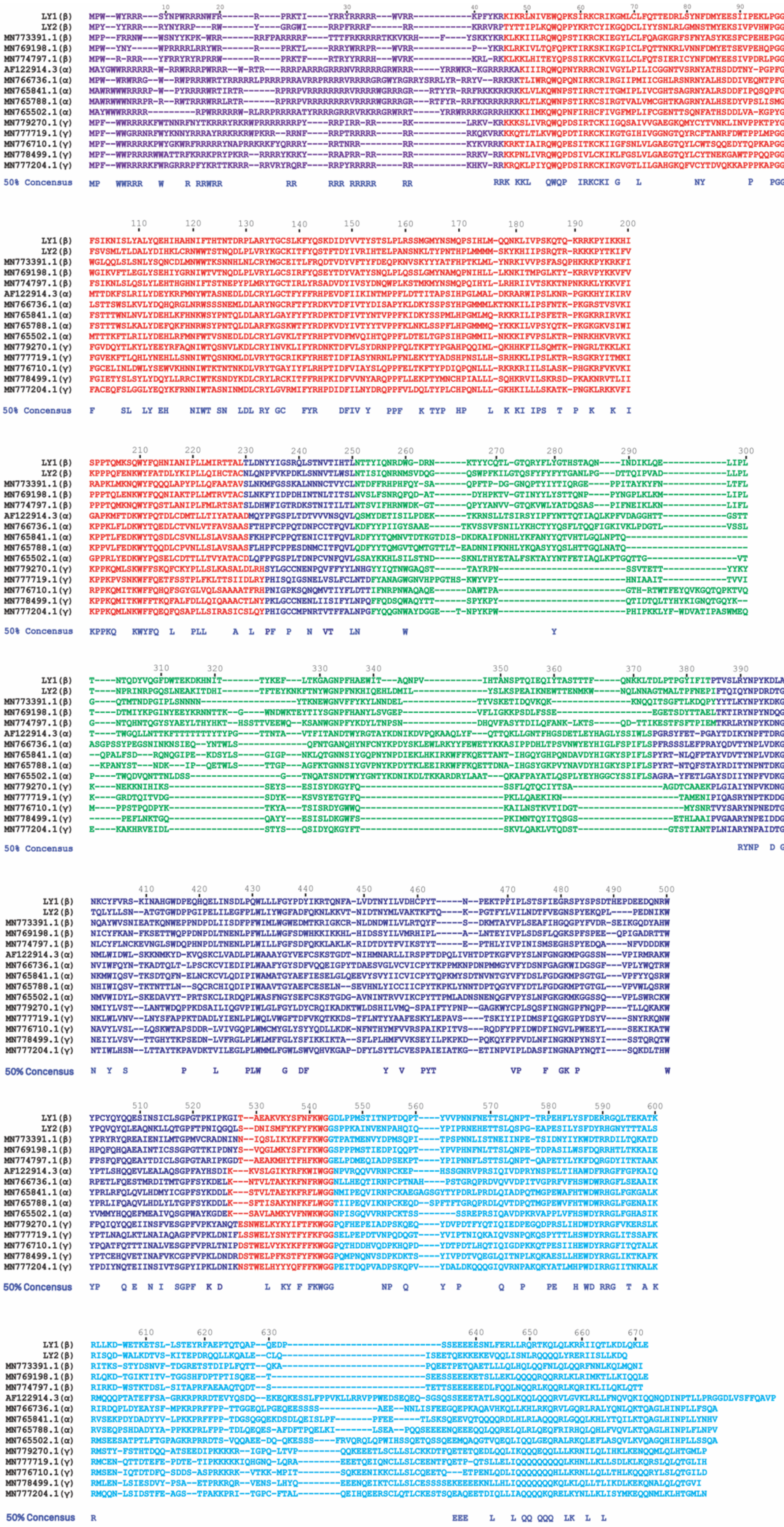
LY1(β)
LY2(β)
MN773391.1(β)
MN769198.1(β)
MN774797.1(β)
AF122914.3(α)
MN766736.1(α)
MN765841.1(α)
MN765788.1(α)
MN765502.1(α)
MN779270.1(γ)
MN777719.1(γ)
MN776710.1(γ)
MN778499.1(γ)
MN777204.1(γ)
50% Concensus

Supplementary Fig. 6. Sequence alignment of 15 published anelloviruses within different genera indicated in parentheses. The residues are colored by domain as in Fig. 1. Below the alignment conserved residues (50% or greater) are indicated.

A

	α-helix	β-sheet	Turns	Unordered
SELCON	60.1%	4.4%	14.6%	21.6%
CONTILL	70.2%	2.2%	7.9%	19.8%
CDSSTR	87.7%	2.2%	3.8%	5.5%
average	72.7%	2.8%	8.8%	15.6%

B

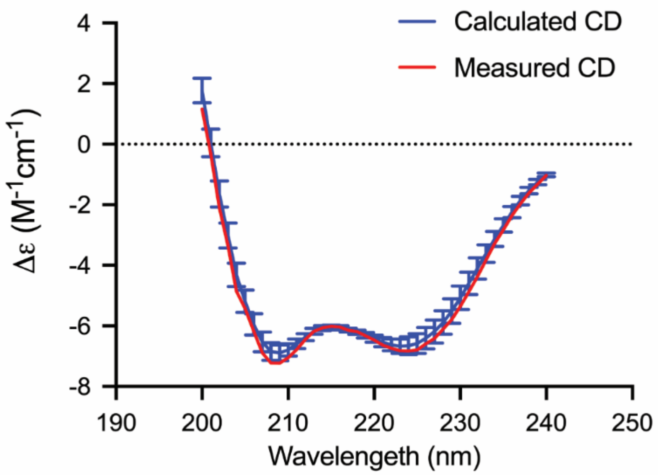

Supplementary Fig. 7. Circular dichroism (CD) of the LY1 C-terminal peptide **A.** Averages of secondary structure fractions estimated by different packages of CDPro. α-helix dominates the secondary structure assignment from the CD spectrum. **B**. An experimental spectrum of the C-terminal peptide (shown in red) overlaid with the calculated and averaged reference set spectra (shown in blue).

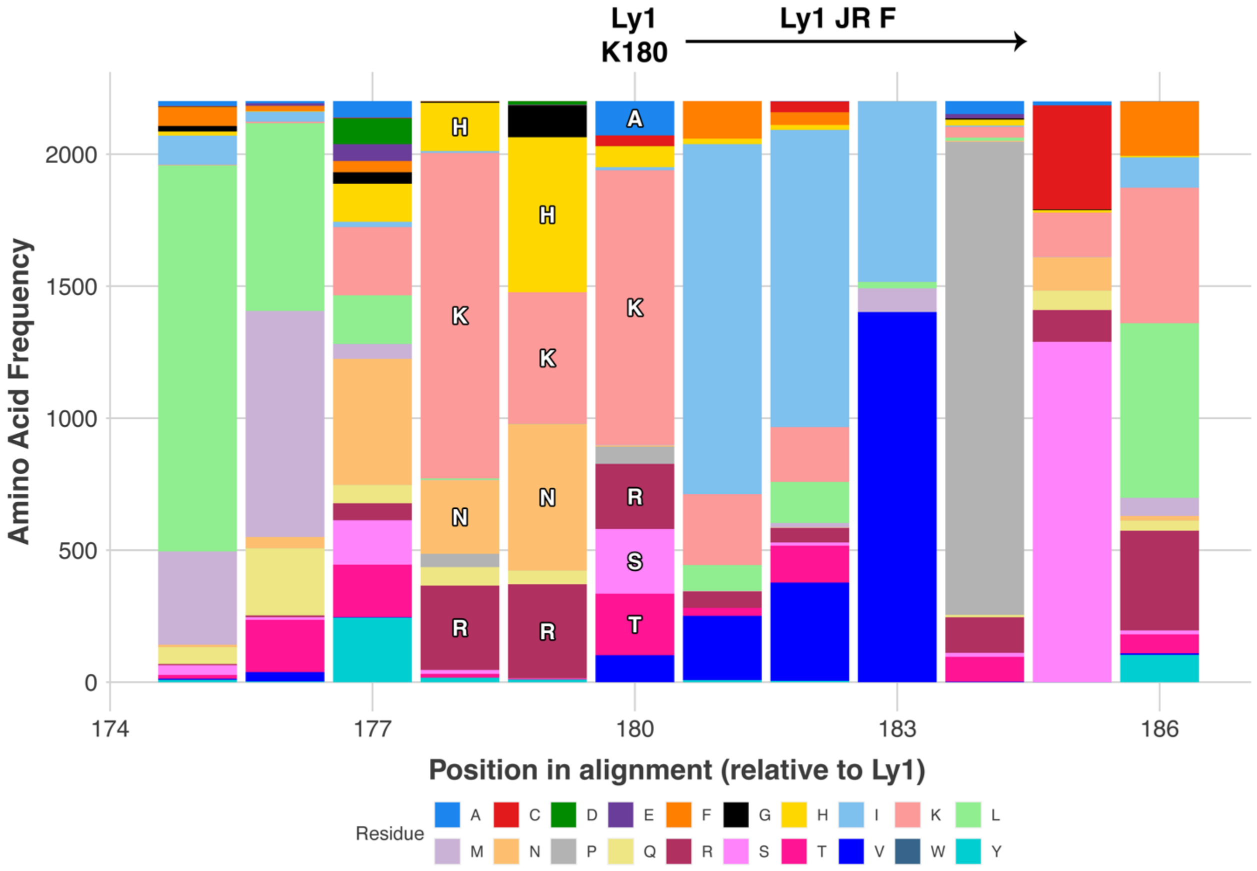

Supplementary Fig. 8. Amino acid distribution for *Betatorqueviruses* around jelly roll strand F. An alignment of 2201 *Betatorquevirus* ORF1 sequences was performed and the amino acid found aligned to LY1 residues 174 to 186 shown (LY1 residue number is indicated on the x-axis). The proportion of each distinct color in each indicates the absolute count of that specific residue present at that position in the alignment. Above the plot the position of LY1 Lys 180 is indicated as well as the position of jelly roll β-strand F. The most common residues aligned to position 178, 179 and 180 are basic or charged residues and are labeled for clarity.

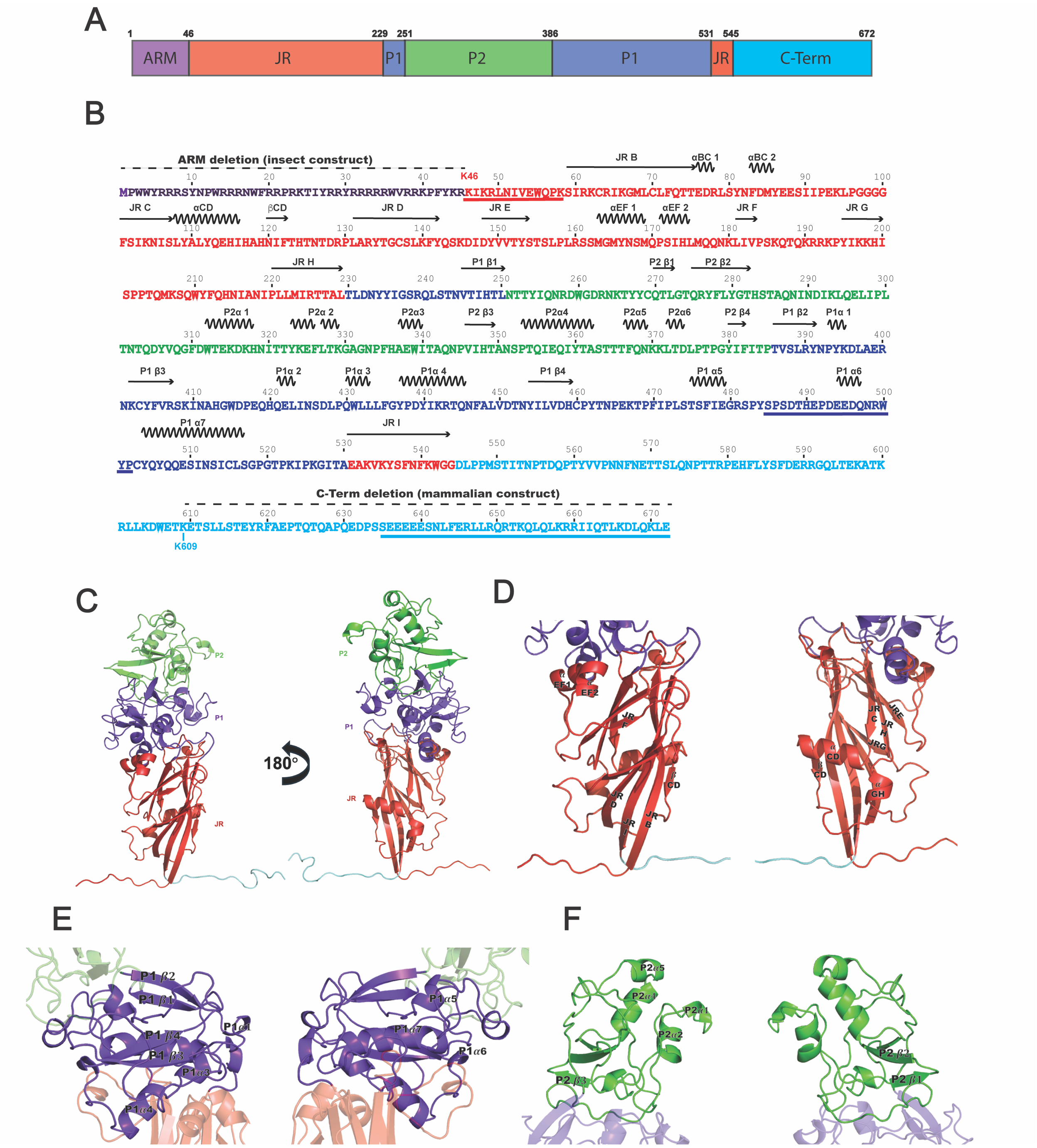

Supplementary Fig. 9: Secondary structural elements marked the monomeric atomic model of LY1 ΔC-term virus-like particle. **A.** Domain organization of ORF1 **B.** Secondary structural elements marked on the ORF1 primary sequence. **C.** Monomer of the ORF1 represented in two orientations. **D. E.** and **F.** Secondary structural elements jelly roll, P1 and P2 domains are highlighted respectively.
